# Proportion and patterns of ocular disorders among under‐five children in Khartoum State, Sudan: A cross‐sectional study

**DOI:** 10.1002/hsr2.1273

**Published:** 2023-05-18

**Authors:** Mohanad K. M. Ibrahim, Jacqueline E. Wolvaardt, Mustafa K. M. Elnimeiri, Hadeel K. M. Ibrahim, Alaa S. A. Mohamed

**Affiliations:** ^1^ Department of Community Medicine, Faculty of Medicine Ibn Sina University Khartoum Sudan; ^2^ Faculty of Health Sciences, School of Health Systems and Public Health University of Pretoria Pretoria South Africa; ^3^ Department of Community Medicine, Faculty of Medicine Alneelain University Khartoum Sudan; ^4^ BSc Clinical Pharmacy Khartoum Sudan; ^5^ BSc Dentistry (BDS) Khartoum Sudan

**Keywords:** epidemiology, eye disease, ocular morbidity, Sudan, under‐five children

## 1 INTRODUCTION

Early childhood is an important period for children's health and development. In addition to physical wellbeing, healthy development of a child is when social, emotional and educational needs are met.^1^

Ocular diseases among children are related to prenatal‐, neonatal‐, or childhood‐underlying causes.^2^ Ocular diseases at a young age might significantly affect children's development and eventually interfere with their quality of life.^3^, ^4^, ^5^, ^6^, ^7^ Visual defects and blindness among young children are a major concern, as they have numerous negative impacts on individuals, the community, and the country. Globally, it is estimated that one child goes blind every minute and 60% die within 1–2 years of becoming blind.^2^ The majority of blind children are in poor African and Asian countries.^2^

Conjunctivitis and refractive errors (REs) are the most common pediatric ocular diseases,^8^ whereas congenital cataracts remain the most common treatable blinding disorder in infancy and childhood. Other common pediatrics ocular diseases include retinopathy of prematurity (ROP),^9^ optic atrophy, advanced glaucoma, anophthalmos, retinal detachment, keratomalacia with perforation, corneal opacities, and myopia.^10^

The detection of ocular diseases is crucial for effective interventions and, therefore, vision screening programs for young children are highly recommended.^11^ However, there are barriers in conducting these programs in developing countries due to scarcity of trained professionals and low enrolment in screening programs. As a result, the majority of young children never get an eye examination. The majority of ocular disorders such as amblyopia, unilateral blindness, and strabismus occur at a young age and is the reason why the American Academy of Pediatrics recommends visual screening programs for children younger than 5 years to ensure early detection and appropriate interventions, which, in turn, result in a better quality of life.^12^

The objective of this research was to study the epidemiological patterns of ocular morbidity among children younger than 5 years old at three tertiary eye hospitals.

## 2 METHODS

A cross‐sectional study was conducted in three tertiary hospitals that have a high turnover in Khartoum State, Sudan. In total, the average number of monthly pediatrics outpatients seen in these hospitals is ~1500 and 200 pediatric eye surgeries are performed per month.

## 3 STUDY POPULATION AND DATA COLLECTION

Patient records of all children younger than 16 years old who attended any of the three hospitals, for any complaint, in 2019 were retrieved (*n* = 10,886) to collect those of under 5 years of age to estimate their proportion and later to study the pattern of ocular disorder. All under‐five children who attended in 2019 were included in this study, whereas no specific exclusion criteria were determined.

The hospitals' electronic records, which include patients' personal and medical information were reviewed. Files were reviewed to identify those who met the inclusion criteria of children younger than 5 years old who had attended any one of these hospitals with any ocular disorder in 2019. Data regarding the demographic profile, as well as types and patterns of ocular morbidities, were then extracted from those who met the inclusion criteria.

## 4 DATA MANAGEMENT AND ANALYSIS

Data sheet was used for data collection. The data were sorted, cleaned, categorized, and summarized on a master sheet and then analyzed using Statistical Package for the Social Sciences version 21.0. The proportions were estimated and patterns of ocular morbidity were identified according to age groups and gender. An Independent‐sample *T* test was conducted to examine any significant difference across patterns of ocular disorders between boys and girls. A *p* < 0.05 was considered as significant. Computerized patient records were never left unattended during data collection and data were directly entered into a password‐protected electronic database.

## 5 ETHICS AND PERMISSIONS

Ethical approval was obtained from Al‐basar Institutional Review Board (B‐IRB‐20‐MR‐012) and permission to access the data was obtained from each of the three hospital's administration. All necessary measures were taken to ensure anonymity of the patients and hospital, and all collected information is confidential.

## 6 RESULTS

**Figure 1:**
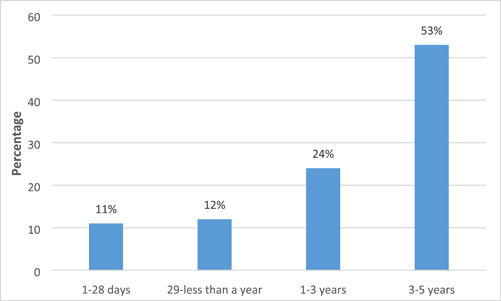
Age distribution of under‐five children.

Under 5‐year‐old children constituted 45% of the total number of children younger than 16 years (*n* = 4899) treated at the sites. Boys to girls ratio was (1.1:1). The age group that was most affected with ocular morbidity was those 3–5 years old (53%, *n* = 2596) (Figure 1).

**Figure 2:**
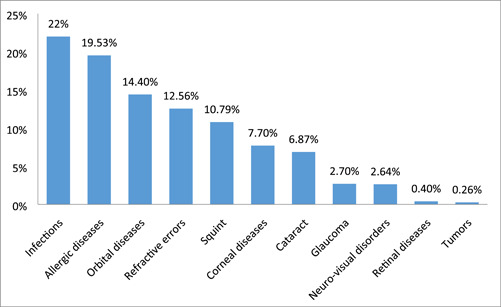
Type of ocular morbidity among under‐five children.

The most common eye disorders affecting patients under 5 years were eye infections (22%), allergic ocular diseases (19.53%), orbital diseases (14.4%), RE (12.56%), squint (10.79%), corneal diseases (7.7%), cataract (6.87%), glaucoma (2.7%), neuro‐visual disorders (2.64%), retinal diseases (0.4%), and tumors (0.26%) (Figure 2).

There was some variation in morbidity according to gender (Table 1). The ocular diseases that affected boys more than girls were as follows: infections, allergic diseases, orbital diseases, corneal diseases, cataract, glaucoma, neuro‐visual disorders, and retinal diseases. The ocular diseases that affected girls more than boys were as follows: RE, squint disorders, and retinal diseases. Boys had a higher overall proportion of ocular disease than girls (*t* = 1.94) (*p* < 0.03).

**Table 1:** Variations in patterns of ocular morbidity according to gender among children under 5 years.

Gender	No of patients	Males	Females	Difference
Infection	2063	53.30%	46.60%	6.7%
Allergic diseases	1831	58.00%	42%	16%
Orbital diseases	1353	50.60%	49.40%	1.2%
Refractive errors	1179	46.30%	53.60%	7.3%
Squint	1012	46.80%	53.10%	6.3%
Corneal diseases	723	54.40%	45.50%	8.9%
Cataract	645	61.50%	38.50%	23%
Glaucoma	254	51.80%	48.10%	3.7%
Neuro‐visual disorders	248	52.00%	48%	4%
Retinal diseases	38	41.60%	58.30%	16.7%
Tumors	25	62.50%	37.50%	25%
Mean		52.62%	47.33%	

*T*‐value = 1.94 and *p* < 0.03.

## 7 DISCUSSION

This research explored the epidemiological patterns of ocular morbidity among children under 5 years of age, who attended any of the three participating tertiary eye hospitals in Khartoum State, Sudan, in 2019.

In this study, children younger than 5 years of age constituted just less than half of the total number of children—younger than 16 years—treated at the three study sites. These results are similar to an Ethiopian study, which reported a prevalence of 32% of ocular morbidity affecting under‐five children.^13^

The study revealed different patterns of ocular diseases from simple to blinding diseases. The most common ocular disease in this study was bacterial, viral and fungal infections. Although most of these infections do not cause permanent damage, some might lead serious visual defects if not treated at an early stage. In most cases, urgent referral to an ophthalmologist for proper diagnosis and treatment is suggested.^14^ Eye infections among young children were the most frequent ocular disorders accounted for about 40% as reported in a similar hospital based study in Karachi.^13^ Allergies were the second most common group of ocular diseases, followed by orbit‐related conditions. In a similar study conducted in Ethiopia, the second commonest eye disorder among young children was ocular allergy.^13^

Orbit‐related conditions comprising the eyelids, tear ducts, and eyeball represented a substantial proportion of ocular disorders in this study. In general, the commonest form of this group of disorders is naso‐lacrimal duct obstruction that can lead to serious complications if left untreated. Infection, which may lead to cellulitis, abscesses and brain involvement if left untreated, remains a major concern. The other form of orbit‐related conditions is eyelid swelling, whether inflammatory or due to tumors.

RE (myopia, hyperopia, and astigmatism) were the fourth commonest of all ocular disorders. Uncorrected RE are associated with abnormal development, including reduced cognitive ability and motor skill in young children.^15^

Some of the children in this study were affected with squint disorders and neuro‐visual disorders. Vision loss can be a consequence of disorders that affect the neural pathway conveying visual input to brain. The presentation and course of vision loss depends on the involved part and the underlying cause.^16^ Cerebral visual impairment (due to damage to the visual pathways in the brain) is the leading cause of severe visual impairment and blindness in children in high‐income countries. It is also an emerging cause in low‐income countries, where a relatively high proportion is attributable to perinatal factors and so potentially avoidable through better perinatal care. Cerebral visual impairment may be missed, because it usually affects children who also have other disabilities such as cerebral palsy or learning difficulties. A community‐based study of cerebral palsy in Bangladesh showed that a third of children had reduced visual acuity and over half had visual perception problems, which adversely affected their quality of life.^17^

This study shows that serious ocular disease that can cause vision loss, including corneal diseases, cataracts, glaucoma, and retinal diseases, were markedly present among the study population. Diseases affecting the cornea are serious, as they can lead to irreversible blindness. Cataracts were frequent ocular disorder in this study. This finding is similar to the proportion of cataract (6.3%) reported by Mehari^18^ in Central Ethiopia. Although cataract is a leading cause of blindness, it can be treated with a relatively simple procedure and the outcome is usually satisfying. Congenital cataracts and ROP affect visual functions in young children and can be prevented or treated. Modalities to prevent, diagnose, and manage these conditions are available. Visual prognosis after cataract surgery in young children has improved considerably, but congenital and infantile cataracts are still responsible for 10% of global childhood blindness and the leading cause of blindness in many countries of Africa.^19^, ^20^

A small percentage of under‐five children in this study were affected by congenital glaucoma. Glaucoma in children is a rare disease with variable incidence across countries and ethnic groups.^21^, ^22^, ^23^, ^24^, ^25^ However, the incidence of glaucoma is higher in the Middle East, including Saudi Arabia, where consanguineous marriages are more prevalent. The estimated incidence of Primary Congenital Glaucoma in Saudi Arabia is 1 per 2500 live births.^26^, ^27^ Glaucoma in young children can lead to significant visual impairment and optic atrophy. The prognosis of glaucoma depends mainly on the time, severity, and age of presentation. Early diagnosis and management are the cornerstone in prevention of visual defects, as late presentation can lead to irreversible and threatening complications.^28^

Few of the under‐five children in this study were diagnosed with tumors, the predominant type being retinoblastoma. Eye tumors are of concern due to the narrow distance between the eyeball and brain, as well as the connection made by the optic nerve and the retina. These features make secondary involvement likely. Retinoblastoma is the most common eye cancer of childhood but it is a relatively rare disease, occurring in ~1 out of every 16,000 to 18,000 live births in the global population. Its incidence is similar across populations and does not vary according to gender, ethnicity, or socioeconomic status. Worldwide, ~8000 children develop retinoblastoma each year, with the vast majority presenting with the disease before the age of 5 years.^29^

The pattern of ocular diseases in this study is similar to what was reported in a study conducted among Egyptian pre‐school children in rural areas, with one exception. Their study reported RE as the most common condition, while this study in it is the fourth most common condition.^30^

The pattern of ocular diseases between the genders showed some variation in this study and the overall finding that boys appear to be disproportionally affected supports the results of a study conducted by Demissie et al.^31^ Gender variations in ocular health are clinically under‐addressed.^32^ Although there are evidence of gender disparities with regard to some ocular diseases prevalent for adult women more than men related to some biological and cultural factors, there are no available data in regard to younger children. The gender variation in this study could be coincidently but still need to be addressed and further studies are needed to clarify any clinical significance.^33^

## 8 CONCLUSION

A considerable number of ocular disorders was noticed among under‐5‐year‐old children. As the majority of these morbidities were preventable or treatable, then early detection through screening programs in addition to health education and promotion would be a valuable contribution for minimization and control.

## AUTHOR CONTRIBUTIONS

**Mohanad K. M. Ibrahim**: Conceptualization; data curation; formal analysis; investigation; methodology; project administration; validation; visualization; writing—original draft. **Jacqueline E. Wolvaardt**: Writing—review & editing. **Mustafa K. M. Elnimeiri**: Writing—review & editing. **Hadeel K. M. Ibrahim**: Writing—review & editing. **Alaa S. A. Mohamed**: Writing—review & editing.

## CONFLICT OF INTEREST STATEMENT

Mohanad K. M. Ibrahim is an Editorial Board member of Health Science Reports and a corresponding author of this article. To minimize bias, they were excluded from all editorial decision‐making related to the acceptance of this article for publication.

## ETHICS STATEMENT

This research was reviewed and approved by Al‐basar Institutional Review Board (B‐IRB‐20‐MR‐012). Ethical permissions were obtained from the hospitals' administration.

## TRANSPARENCY STATEMENT

The lead author Mohanad Kamaleldin Mahmoud Ibrahim affirms that this manuscript is an honest, accurate, and transparent account of the study being reported; that no important aspects of the study have been omitted; and that any discrepancies from the study as planned (and, if relevant, registered) have been explained.

## Data Availability

The data sets used and/or analyzed during the current study are available from the corresponding author on reasonable request.
